# Ellagic acid and cilostazol ameliorate amikacin-induced nephrotoxicity in rats by downregulating oxidative stress, inflammation, and apoptosis

**DOI:** 10.1371/journal.pone.0271591

**Published:** 2022-07-18

**Authors:** Zeinab Mahmoud Saeed, Monira Ismail Khattab, Nadia Esmat Khorshid, Amal Elsayed Salem

**Affiliations:** Clinical Pharmacology Department, Faculty of Medicine, Zagazig University, Zagazig, Egypt; Universidad Autónoma de Coahuila, MEXICO

## Abstract

Amikacin (AK) has the largest spectrum of aminoglycosides. However, its use is constrained because of nephrotoxicity and ototoxicity. Ellagic acid (EA) is a polyphenol present in plants. It has antioxidant, anticarcinogenic, and antimutagenic characteristics. Cilostazol (CTZ) is a phosphodiesterase Ш inhibitor, it is a potent vasodilator and antiplatelet drug. CTZ has an inhibitory effect on reactive oxygen species and superoxide generation in addition to hydroxyl radicals scavenging action. This study determines whether EA and cilostazol have a protective effect against AK-induced nephrotoxicity. Forty-nine rats were divided into seven equal groups: control normal; AK 400 mg/kg; EA 10 mg/kg; CTZ 10 mg/kg; AK 400 mg/kg plus EA 10 mg/kg; AK 400 mg/kg plus CTZ 10 mg/kg; AK 400 mg/kg plus EA 10 mg/kg and CTZ 10 mg/kg. For seven days, drugs were administered using gavage one hour before intramuscular injection of AK. Twenty-four hours after the last AK dosage, blood samples were collected to determine blood urea nitrogen and creatinine levels. Kidneys were removed for histopathological examination and measurement of: malondialdehyde (MDA), catalase (CAT), decreased glutathione (GSH), superoxide dismutase (SOD), interleukin 6 (IL6), tumor necrosis factor-alpha (TNFα), nuclear factor kappa B (NFκB), and Bcl-2 associated x protein (BAX). AK caused kidney damage, inflammatory mediator elevation, and oxidative stress and apoptotic markers. Rats receiving EA or CTZ indicated significant improvement in kidney function, decrease in oxidative stress and inflammation through NF-kB down-regulation and BAX expression. The combination of EA and CTZ showed a synergistic effect. In conclusion, EA and CTZ might play a beneficial role in preventing nephrotoxicity induced by AK partially by inhibition of tissue inflammation and apoptosis.

## Introduction

Nephrotoxicity is defined as a 50% increase in serum creatinine or a 50% decrease in creatinine clearance and an increase in blood urea nitrogen. Drugs like aminoglycosides, chemotherapeutic agents, angiotensin-converting enzyme inhibitors, non-steroidal anti-inflammatory drugs, angiotensin receptor blockers, vancomycin, amphotericin B and chemicals as well as radio contrast cause 20% of nephrotoxicity [1]. The mechanisms underlying nephrotoxic-induced renal cell death and renal diseases are surprisingly similar. ATP depletion, oxidative stress, proximal tubule cell death and loss of the brush border membrane, and cell polarity are all involved in ischemia-induced acute kidney injury (AKI) [2]. AKI induced by cancer chemotherapeutic, such as cisplatin, alternatively, includes oxidative stress, proximal tubule cell death, and loss of the brush border membrane and polarity [3]. Increased oxidative stress, ATP loss, and proximal tubule cell death are all common manifestations of nephrotoxicity caused by contrast media, also known to affect glomerular function and renal blood flow [4].

Amikacin (AK) has the broadest spectrum and the least resistance of all aminoglycosides. AK is preferred owing to its advantageous characteristics, including rapid and robust bactericidal activity, synergy with β-lactam antibiotics, low cost, chemical stability, and low resistance; however, its use is limited because of the risk of nephrotoxicity and ototoxicity [5]. Because AK is not metabolized in the body and is eliminated in large amounts in the urine, it builds up in the proximal convoluted tubules, causing free radical manufacture and renal damage [6]. Several mechanisms, such as inflammation, blockage of transporters, production of oxidative stress, and decreased renal blood flow, are involved in amikacin-induced renal damage [7].

Ellagic acid (EA) is a polyphenolic compound naturally found in plants. Several studies have indicated that EA has antioxidant, anti-apoptotic, and anticarcinogenic qualities. This antioxidant action of this compound is determined by its chemical structure, precisely the number of hydroxyl groups and their ability to boost the stability of the phenoxyl radicals [8]. EA reduces the expression of proinflammatory and profibrogenic cytokines, such as tumor necrosis factor-alpha (TNFα), transforming growth factor-beta (TGFβ), and many interleukins involved in alcohol-induced inflammation and fibrosis [9].

Cilostazol (CTZ) is a strong antiplatelet and vasodilator that is a specific PDE III inhibitor. It increases intracellular cyclic adenosine monophosphate (cAMP) levels [10]. It also increases cyclic guanosine monophosphate (cGMP) [11]. CTZ prevents oxidative stress by activating redox defense systems through increased expression of phosphoinositide 3-kinase/protein kinase B (PI3K/Akt) and nuclear factor erythroid 2-related factor/heme oxygenase-1 (Nrf2/HO-1) mRNAs, resulting in oxidative stress reduction and restoration of mitochondrial dysfunction [12].

This study determines possible nephroprotective effects of EA, CTZ, their combination, and the underlying mechanism of renal tubular necrosis induced by AK.

## Materials and methods

### Drugs and reagents

Amikacin (Amikacin®) 500 mg/2ml vial (Amoun Pharmaceutical Co., EL-obour city, Cairo, Egypt). All other drugs were bought from Sigma Aldrich (St. Louis, MO).

### Experimental animals and ethical statement

**Fig 1:**
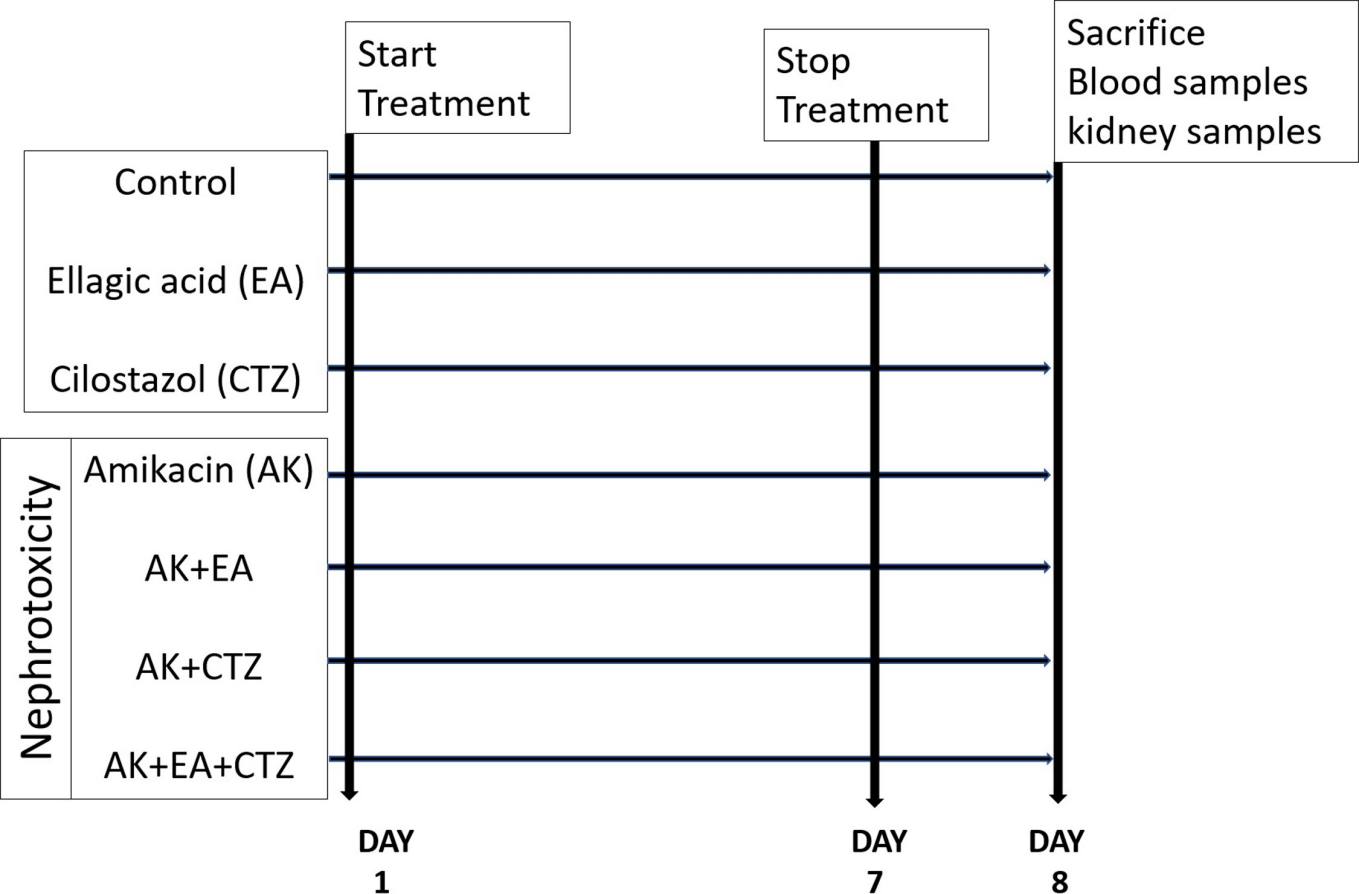
Experimental design.

Forty-nine male albino rats weighing 150–180-g were obtained from Zagazig University Faculty of Veterinary Medicine. The animals were placed in hygienic and standard environmental conditions (25 ± 2°C) and 12 h light/dark cycle. They were given access to water and food *ad-libitum*. The study was approved by Zagazig University’s local animal Ethical Committee. The approval number is ZU-IACUC/3/F/57/2019. National Institutes of Health’s guidelines (USA) were followed throughout the experiment. Rats were randomly divided into seven groups; each group having seven rats as follows:

Group 1: non-treated (control normal);
Group 2: received AK 400 mg/kg, intramuscular injection once daily for seven days for induction of experimental nephrotoxicity as described by [13].
Groups 3: received EA 10 mg/kg; dissolved in 1 ml distilled water and given by oral gavage according to [14].
Groups 4: received CTZ 10 mg/kg; dissolved in 1 ml distilled water and given by oral gavage according to [15]
Group 5: received EA 10 mg/kg, orally by gavage one hour before intramuscular injection of AK 400 mg/kg.
Group 6: received CTZ 10 mg/kg, orally by gavage one hour before intramuscular injection of AK 400 mg/kg.
Group 7: received EA 10 mg/kg plus CTZ 10 mg/kg, orally by gavage one hour before intramuscular injection of AK 400 mg/kg (**Fig 1**).

### Collection of blood and renal samples

On the 8^th^ day of the experiment, the animals were anesthetized using a 50 mg/kg intraperitoneal injection of sodium pentobarbital for sacrifaction. The blood samples from the retro-orbital plexus of veins were collected using microcapillary tubes. These samples were centrifuged at 3000 × g for ten minutes to separate the serum for determining serum BUN and creatinine concentrations. The left kidneys were dissected for histopathological examination and biochemical estimation of malondialdehyde (MDA), reduced glutathione (GSH), superoxide dismutase (SOD), catalase (CAT), nuclear factor kappa B (NFκB), interleukin 6 (IL6), tumor necrosis factor-alpha (TNFα), and Bcl-2 associated x protein (BAX). The remaining kidneys were frozen at −80°C and ice-cold 0.05 M phosphate buffer pH 7.4 until used.

### Biochemical analysis

#### Determination of BUN and creatinine

Using kits bought from Spinreact (Gerona, Spain) and following the manufacturer procedure.

#### Estimation of lipid peroxidation marker

MDA levels in kidney homogenates were measured by spectrophotometry. Kit reagents obtained from ZeptoMatrix corporation, Bufflo, united states (catalog No: 0801192). kidney samples were homogenized in ice-cold 50 mM potassium phosphate buffer (pH 7.5), centrifuged for 15 min at 4°C 12,000 × g then the supernatant was obtained. MDA in the supernatant can generate a colorful complex with thiobarbituric acid, which was absorbed maximally at 535 nm [16].

#### Estimation of the antioxidant parameters; GSH, CAT, and SOD

Colorimetric kits were obtained from Dokki Biodiagnostic Company in Giza, Egypt. Measurements were carried out on reduced glutathione levels using the colorimetric method based on the reduction of 5,5`dithiobis (2-nitrobenzoic acid) (DTNB) with glutathione (GSH) to obtain a yellow compound. The decreased chromogen is directly proportional to GSH concentration, and its absorbance can be measured at 405 nm [17]. CAT was measured following the method performed by [18]. SOD activity was determined using the method described by [19].

#### Quantitative estimation of tumor necrosis factor-alpha (TNF-α) and interleukin 6 (IL6) concentration in renal tissue

They were analyzed using USCN Life Science Inc. ELISA kits. According to the manufacturers’ protocol, the competitive inhibition enzyme immunoassay technique was used in this assay.

#### Estimation of Bcl-2 associated x protein (Bax) and nuclear factor kappa B (NFκB) in renal tissue

It was determined using quantitative real-time PCR after total RNA was isolated according to the manufacturer’s instructions using the Qiagen tissue extraction kit (Qiagen, USA). Using a high-capacity cDNA reverse transcription kit (Fermentas, USA), total RNA was converted to cDNA. Then, using Applied Biosystems with Step One TM software version 3.1 (USA), amplification and analysis of real-time qPCR product were conducted. The primer sequence of the gene under study include:

BAX: Forward primer:5’-CCCTGTGCACTAAAGTGCCC-3.
Reverse primer: 5’-CTTCTTCACGATGGTGAGCG-3
NFκB: Forward primer: 5’-CATTGAGGTGTATTTCACGG -3
Reverse primer: 5’-GGCAAGTGGCCATTGTGTTC -3

### Histopathological studies

The kidneys were quickly extracted and opened. The specimens were fixed in 10% formalin, sectioned into 5 mm thick paraffin blocks, and hematoxylin as well as eosin stains (H&E) were used for light microscopy [20].

### Statistical analysis

To compare all groups, a one-way analysis of variance (ANOVA) was conducted, while to compare between every two groups, the post-hoc Turkeyʼs test was used. All data are expressed as mean ± SEM. A p-value less than 0.05 is considered significant. Computer analysis of the obtained data was conducted using the Statistical Package for Social Services version 25 (SPSS).

## Results

### Effect of ellagic acid, cilostazol and their combination on renal function

**Fig 2:**
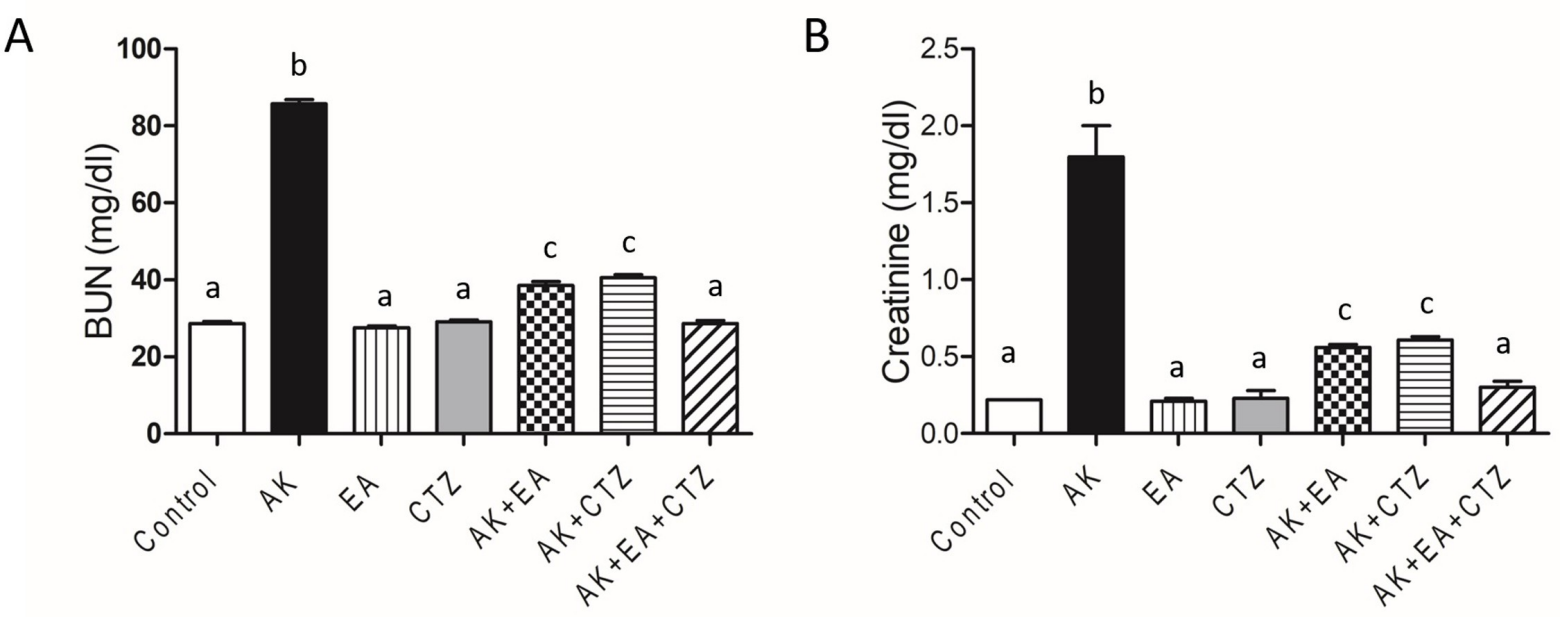
The effect of ellagic acid, cilostazol, and their combination on renal function. Graphical presentation of serum BUN (blood urea nitrogen) (A) and creatinine (B). Ellagic acid (EA) 10 mg/kg, cilostazol (CTZ) 10 mg/kg and their combination were administered one hour before intramuscular injection of amikacin 400 mg/kg for seven days. Groups were compared using one-way ANOVA and post-hoc Turkeyʼs test. Values are presented as mean ± SE (n = 7). Values without common small letters are significantly different (*p* < 0.05).

AK 400 mg/kg significantly increased BUN and creatinine levels compared with the normal control group. EA 10 mg/kg or CTZ 10 mg/kg alone produced a non-significant reduction in both parameters in relation to the normal control group. AK plus EA and AK plus CTZ significantly reduced BUN and creatinine compared with the AK group. AK plus EA and CTZ significantly reduced both parameters than each drug alone (**Fig 2**).

### Effect of ellagic acid, cilostazol, and their combination on oxidative stress markers

**Fig 3:**
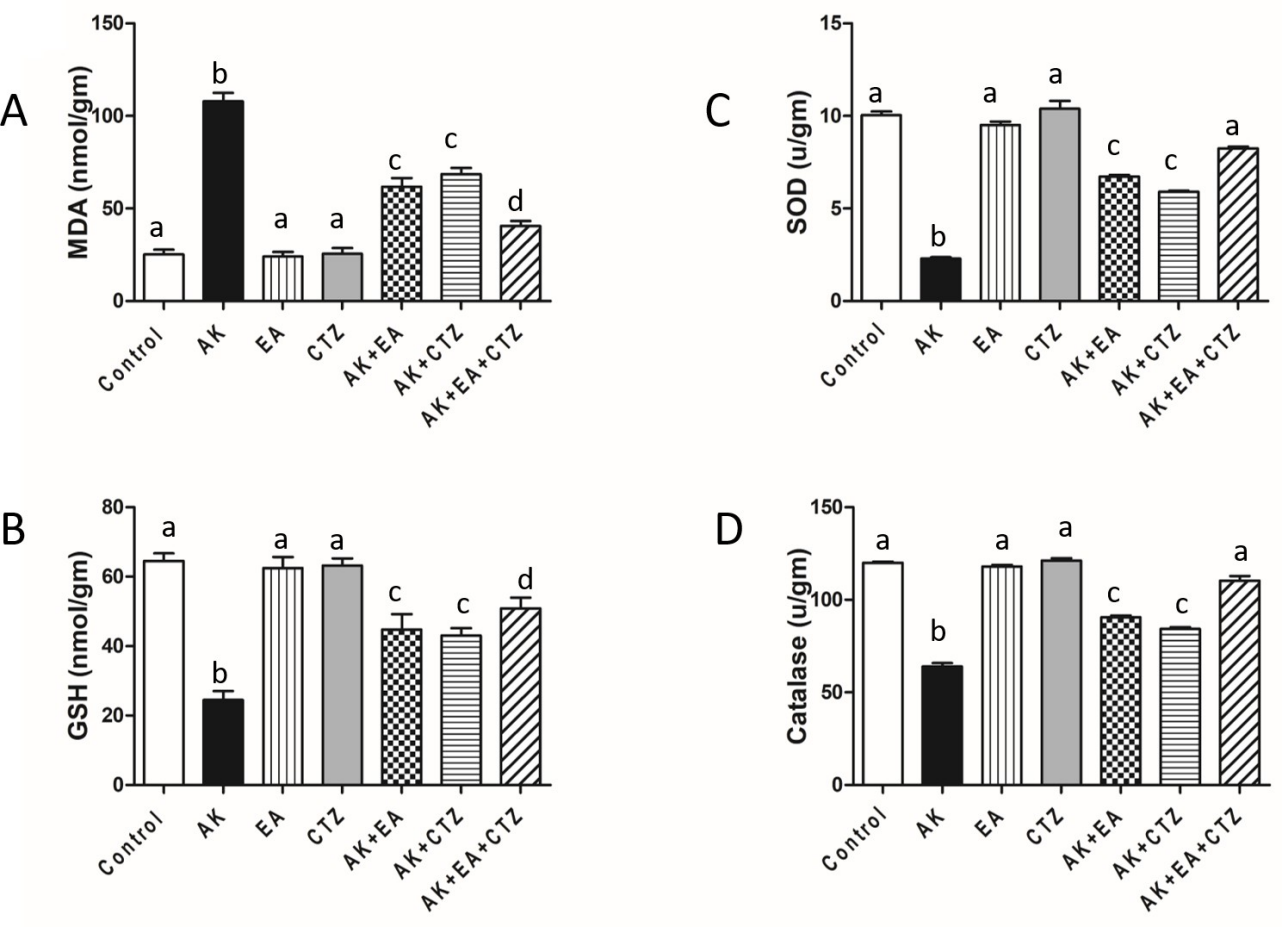
Effect of ellagic acid, cilostazol, and their combination on oxidative stress markers in renal tissue. Quantitative analysis of malondialdehyde (MDA) (A), reduced glutathione (GSH) (B), superoxide dismutase (SOD) (C), catalase (CAT) (D) in the renal tissue. Ellagic acid (EA) 10 mg/kg, cilostazol (CTZ) 10 mg/kg and their combination were administered one hour before intramuscular injection of amikacin 400 mg/kg for seven days. Groups were compared using one-way ANOVA and post-hoc Turkeyʼs test. Values are indicated as mean ± SE (n = 7). Values without common small letters are significantly different (*p* < 0.05).

AK 400 mg/kg significantly increased MDA levels in the renal tissue and caused a significant reduction of CAT, SOD, and GSH in renal tissue compared to the control group. EA10 mg/kg or CTZ 10 mg/kg alone produced non-significant results concerning the normal control group. AK plus EA and AK plus CTZ significantly decreased MDA and significantly increased GSH, SOD, and CAT in renal tissue concerning the AK group. AK plus EA and CTZ produced a more significant MDA reduction and a more significant rise of GSH, SOD, and CAT than each drug alone (**Fig 3**).

### Effect of ellagic acid, cilostazol, and their combinations on inflammatory markers

**Fig 4:**
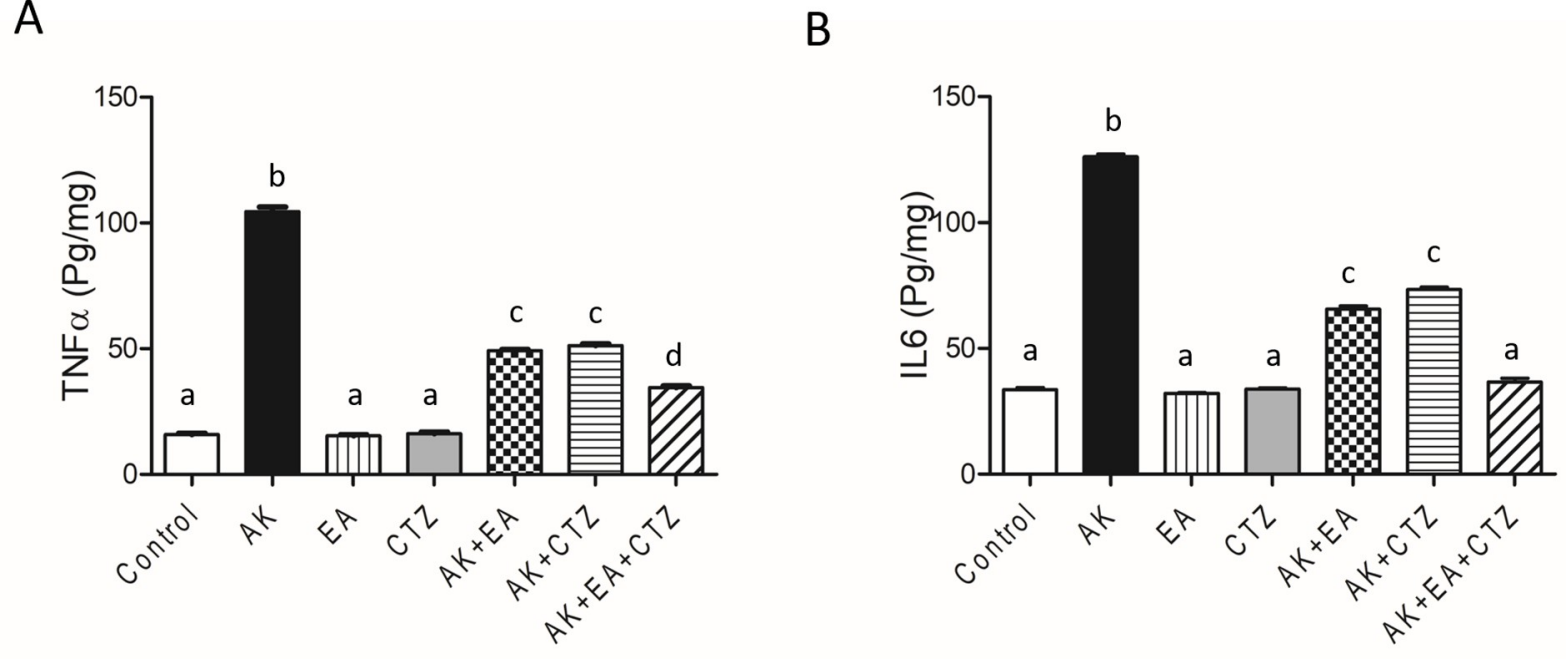
The effect of ellagic acid, cilostazol, and their combinations on inflammatory markers. Quantitative analysis of tumor necrosis factor-alpha (TNF-α) (A) and interleukin 6 (IL6) (B) in the renal tissue. Ellagic acid (EA) 10 mg/kg, cilostazol (CTZ) 10 mg/kg and their combination were administered one hour before intramuscular injection of amikacin 400 mg/kg for seven days. Groups were compared using one-way ANOVA and post-hoc Turkeyʼs test. Values are presented as mean ± SE (n = 7). Values without common small letters are significantly different (*p* < 0.05).

AK 400-mg/kg produced a significant increase in renal tissue TNFα and IL6 compared to the normal control group. EA 10 mg/kg or CTZ 10 mg/kg alone produced non-significant results concerning the control group. AK plus EA and AK plus CTZ produced a significant reduction in TNFα and IL6 in renal tissue compared to the AK group. AK plus EA and CTZ produced a more significant decrease in TNFα and IL6 than each drug alone (**Fig 4**).

### Effect of ellagic acid, cilostazol, and their combination on apoptotic markers

**Fig 5:**
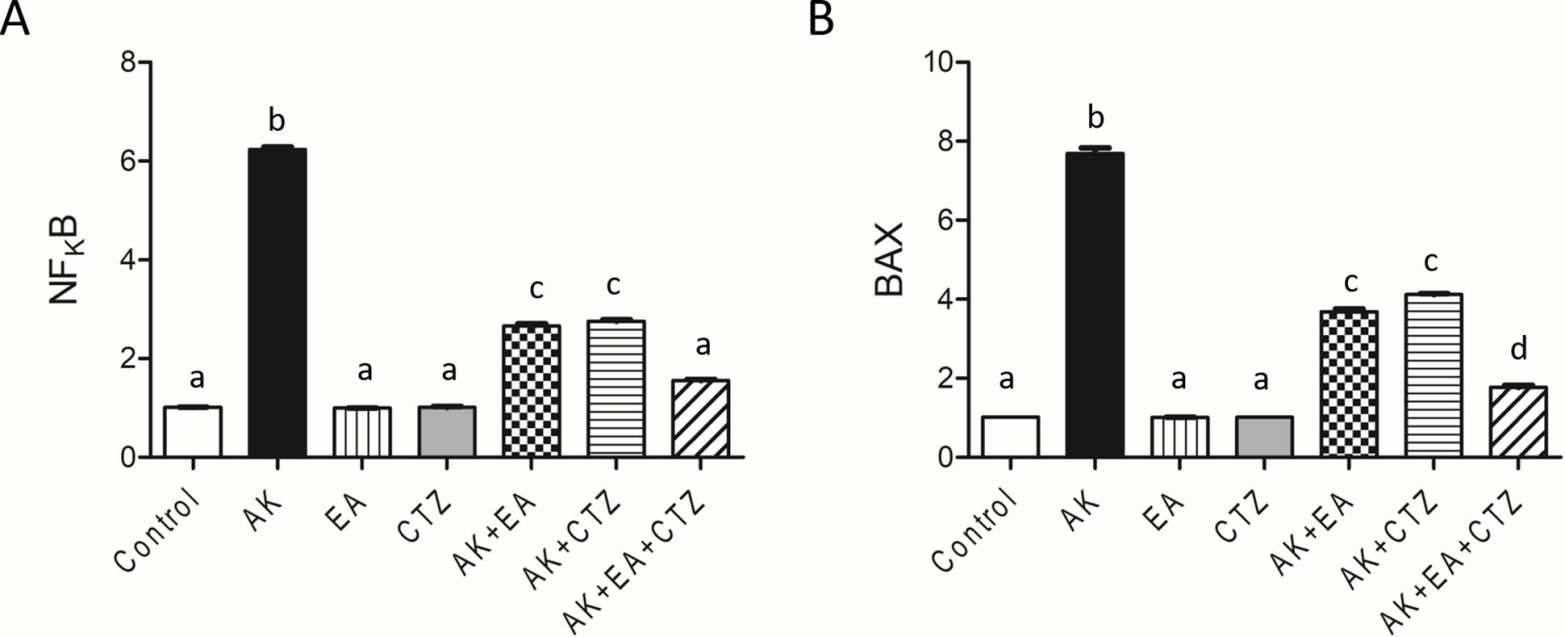
The effect of ellagic acid, cilostazol, and their combination on apoptotic markers. Quantitative analysis of Bcl-2 associated x protein (BAX) and nuclear factor kappa B (NFκB) in renal tissue. Ellagic acid (EA) 10 mg/kg, cilostazol (CTZ) 10 mg/kg and their combination were administered one hour before intramuscular injection of amikacin 400 mg/kg for seven days. Groups were compared using one-way ANOVA and post-hoc Turkeyʼs test. Values are presented as mean ± SE (n = 7). Values without common small letters are significantly different (*p* < 0.05).

AK 400 mg/kg resulted in a significant increase in NFκB and BAX expression in renal tissue compared with the normal control group. EA 10 mg/kg or CTZ 10 mg/kg alone produced a non-significant result concerning the normal control group, while AK plus EA and AK plus CTZ showed a significant decrease in NFκB and BAX expression concerning the AK group. AK plus EA and CTZ produced a more significant NFκB reduction and BAX expression in renal tissue than each drug alone (**Fig 5**).

### Effect of ellagic acid, cilostazol, and their combination on the structure of the renal cortex

Histopathological findings exhibited the normal structure of the renal cortex, tubules, and glomeruli [A]. After nephrotoxicity was induced by amikacin, the kidney exhibited karyolysis, loss of the outer basement membrane of tubules, and accumulation of necrotic material in the lumen [B].

**Fig 6:**
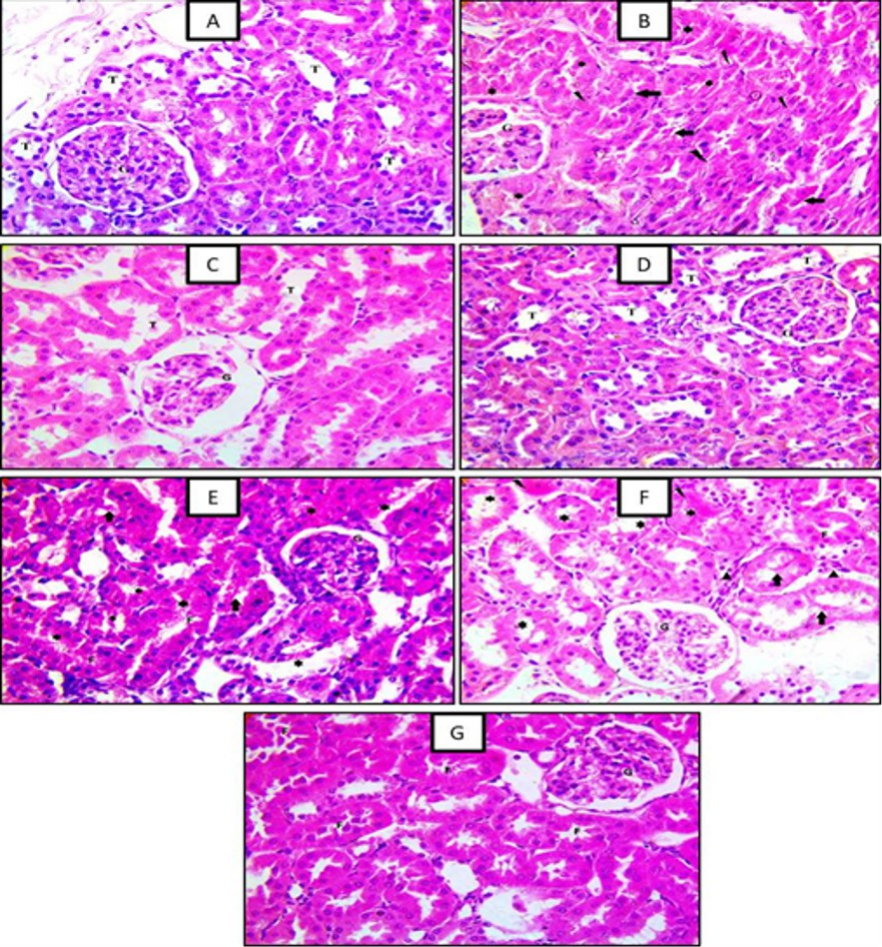
Effect of ellagic acid, cilostazol, and their combination on the structure of the renal cortex. A photomicrograph (H&E stain x400) of renal tissue showing: (A) a normal structure of the renal cortex, tubules (T) and glomeruli (G); control normal, (B) Amikacin (AK), (C) ellagic acid (EA), (D) cilostazol (CTZ), (E) AK + EA, (F) AK + CTZ and (G) AK+EA+CTZ. Ellagic acid (EA) 10 mg/kg, cilostazol (CTZ) 10 mg/kg and their combination were administered one hour before intramuscular injection of amikacin 400 mg/kg for seven days.

EA 10 mg/kg produced no changes in normal kidney structure [C]. Also, CTZ 10 mg/kg produced no changes in normal kidney structure [D]. The administration of EA 10-mg/kg or CTZ 10 mg/kg one hour before AK as a prophylactic agent exhibited an improvement of AK-induced nephrotoxicity in reducing the percentage of the area of inflammation, eosinophilia, and necrosis [E]&[F] respectively. Administration EA plus CTZ one hour before AK as a prophylactic agent showed more reduction in the percentage of the area of inflammation, eosinophilia, and necrosis [G] than each drug alone **(Fig 6)**.

## Discussion

AKI occurs in 20–30% of children exposed to aminoglycosides [21]. This study investigated the renoprotective effect of EA alone, CTZ alone, and their combination.

The results of the current work showed that AK 400 mg/kg significantly increased serum BUN and creatinine levels. These results agree with Hlail and colleagues [1], who demonstrated than intraperitoneal AK 120 mg/kg injection for 14 d produced a significant increase in serum creatinine and urea levels. Also, previous studies found that i.m injection of AK 100 mg/kg for seven days produced a significant rise in creatinine, uric acid, and urea [5].

Multiple pathophysiological effects of AK-induced kidney damage include the creation of reactive oxygen and nitrogen species and stimulation of apoptosis, as AK forms a complex with mitochondrial Fe2+, causing the development of free radicals. These free radicals and reactive species are essential in drug-induced renal impairment and BUN and creatinine increase [7].

Antioxidant enzymes like SOD and CAT are essential for cellular antioxidative defense. SOD catalyzes the formation of hydrogen peroxide (H_2_O_2_) by superoxide radical dismutation [22]. MDA is a lipid peroxidation end product that can be used as a biological biomarker to describe the degree of oxidative stress [23]. GSH acts as a potent electron donor acting against free radicals. With the aid of glutathione peroxidase enzymes, GSH can degrade H_2_O_2_ to H_2_O [24].

Also, this work indicated that AK 400 mg/kg produced a significant reduction in the antioxidant parameters; GSH, SOD, and CAT, and a significant increase in oxidation parameter; MDA, in renal tissue. These results are following Abdel-Daim and colleagues [5] who reported a significant elevation of MDA and a significant reduction of SOD, CAT, and GSH caused by AK 100 mg/kg.

AK is not metabolized in the body and is primarily eliminated in the urine. As a result, it accumulates in proximal tubules and glomeruli, leading to the activation of renin-angiotensin-aldosterone system, lowering the glomerular filtration rate and increasing the production of platelet-activating factor, reactive oxygen species (ROS), and vasoconstrictors [25]. The excessive ROS production causes oxidative stress, which causes significant interconnected disturbances in cellular metabolism, such as protein and nucleic acid structure changes, DNA damage, apoptosis induction, elevation in intracellular free calcium, damage to membrane ion transport, and cell damage from lipid peroxidation [1].

TNF is a proinflammatory cytokine formed by macrophages and monocytes and can activate neutrophils and lymphocytes, enhancing vascular endothelial cell permeability, and triggering the production and release of other cytokines. It acts on tumor necrosis factor receptor 1 (TNFR1) and 2 (TNFR2). TNFR1 mediate inflammation and increases fibroblast proliferation by activating nuclear factor (NF). TNFR2 contributes to cell migration, regeneration, proliferation, and TNF1-mediated apoptosis regulation. TNFα may stimulate the NF-B pathway, which regulates the transcription and production of inflammatory mediators. This is a vicious cycle that exacerbates inflammatory reactions [26].

The results of this work proved that AK 400 mg/kg produced a significant increase in NFκB, TNFα, and IL6 in relation to the normal control group. Ozbek *et al*. (2009) agreed with these results and stated that intraperitoneal injection of gentamycin 100 mg/kg significantly increased NFκB expression in renal tissue [27].

AK-induced nephrotoxicity could be due to up-regulation of TNF-α expression or due to AK-induced oxidative stress, which induces oxygen-containing derivatives and cytokine production, which function as a second messenger for activating NF-B, resulting in the transcription of cytokines, growth factors, and extracellular matrix proteins [5]

This work indicated that AK 400 mg/kg significantly increased BAX expression in renal tissue. Helmy *et al*. (2020) agreed with this result and showed that AK 1.2 g/kg single intraperitoneal injection increased BAX expression in renal tissue [28].

Aminoglycosides can cause apoptosis in the kidney by increasing the content of cytosolic BAX protein, which activates the mitochondrial pathway of apoptosis, it includes caspase-9 activation as an initiator, caspase-3 activation as an effector, and DNase activation, leading to DNA fragmentation and apoptosis [29].

In this study, oral administration of EA 10 mg/kg one hour before AK significantly reduced BUN and creatinine. These findings support those of [14], who reported that EA 10 mg/kg significantly reduced urea and creatinine in gentamycin 100 mg/kg induced nephrotoxicity. Ateşşahín *et al*. (2007) [30] reported that EA 10 mg/kg significantly reduced urea and creatinine in nephrotoxicity induced by intraperitoneal injection of cisplatin 7 mg/kg. The improvement in RBF and GFR could explain EA’s favorable effect in improving kidney function tests and lowering creatinine and BUN levels [31].

In this study, EA 10 mg/kg orally one hour before AK produced a significant reduction of oxidation parameter; MDA, and a significant increase in the antioxidant parameters; GSH, CAT, and SOD in renal tissue in relation to the AK group. A previous study [14] agreed with these results as they reported that EA 10 mg/kg induced a preventive effect on nephrotoxicity caused by gentamycin as it increased SOD, CAT, and GSH levels. Also, Bhattacharjee *et al*. (2021) reported that oral administration of EA 25, 50 mg/kg, orally for two months showed a preventive effect on nephrotoxicity caused by lead by increasing CAT, SOD, GSH, and reducing MDA compared with the control nephrotoxic group [32].

The ability of EA to scavenge free radicals has been related to its intrinsic antioxidant activity. This is since it can transfer the phenolic H-atom to a free radical. Lactone systems and EA hydroxyl groups can create hydrogen bonds and act as hydrogen donors and electron acceptors. As a result, EA can participate in antioxidant redox reactions, resulting in a highly efficient free radical scavenger [33].

Oxidative stress has been shown to be decreased by EA through modulation of several mechanisms. These involve antioxidant response activation through Nrf2, suppression of cytokines, such as IL1, IL6, TNF, and cyclooxygenase 2 (COX-2) through NF-kB, and cell survival or apoptosis control through NF-kB [34]. EA is classed as a multiple-function antioxidant since it exerts its beneficial effect through both primary and secondary ways [35].

EA 10 mg/kg orally one hour before AK significantly reduced TNFα, IL6, and NFκB expression in this work. These findings are consistent with that of Marn *et al*. (2013), who suggested that EA reduced NF-B, IL-6, and TNF levels compared to the control group in mice with ulcerative colitis [36]. EA inhibits inflammation through modulating the NF-B signaling pathway [37]. These findings are consistent with Cornélio Favarin *et al*. (2013), who discovered that EA 10 mg/kg increased the anti-inflammatory cytokine IL-10 and decreased the proinflammatory cytokine IL-6 in bronchoalveolar lavage fluid [38]. EA decreases toll-like receptor 4 (TLR4) and high mobility group protein 1 (HMGB1) in the kidney tissue by cutting down TLR4 downstream protein leading to reduction in inflammatory factors [26].

In this study, EA 10 mg/kg orally one hour before AK reduced BAX expression in renal tissue. A previous study [14] agreed with this finding and reported that EA 10 mg/kg reduced gentamycin-induced nephrotoxicity in rats by increasing Bcl2/BAX ratio and decreasing Caspase- 3. It is one of the main executors of apoptosis.

EA’s antioxidant and anti-apoptotic qualities may be attributed to the increased SIRT1 expression in renal tissues [39]. SIRT1 (sirtuin1) is the mammalian homolog of the yeast Sir2 (silent information regulator 2). It protects against oxidative stress by deacetylating forkhead box O (FOXO) and tumor suppressor protein (p53). SIRT1 deacetylates p53 and FOXO, resulting in transcriptional activities suppression and loss of stress-induced apoptosis [40]. FOXOs also contribute to the viability of cells through the transactivation of enzymes that detoxify ROS, such as SOD2/MnSOD and CAT [39].

Also, this study indicated that oral administration of CTZ 10 mg/kg one hour before AK significantly decreased BUN and creatinine. These findings support a previous study [15] which demonstrated that administration of CTZ 10 mg/kg once daily for eight days reduced creatinine, urea, and uric acid levels in the nephrotoxicity induced by gentamycin. Also, Gokce *et al*. (2012) reported concomitant use of CTZ 10 mg/kg, orally with cyclosporine reduced urea and creatinine level [41].

This work showed that oral administration of CTZ 10 mg/kg one hour before AK produced a significant decrease in oxidation parameter; MDA and a significant increase in the antioxidant parameters; GSH, CAT, and SOD in renal tissue in relation to the AK group. These results agree with that of Gokce *et al*. (2012), who reported that administration of CTZ 10 mg/kg for seven days eases cyclosporine-induced nephrotoxicity by decreasing MDA and increasing SOD and CAT activity [41]. CTZ prevents oxidative stress by activating redox defense systems through increased expression of PI3K/Akt and Nrf2/HO-1 mRNAs, resulting in oxidative stress reduction and restoration of mitochondrial dysfunction [12].

In this study, oral administration of CTZ 10 mg/kg decreased TNFα, IL6, and NFκB expression in renal tissue. These results are according to Hermes *et al*. (2016), who reported that oral administration of CTZ 100 mg/kg for 14 days decreased TNFα and NFκB in dystrophic diaphragm muscle [42]. Also, Sakamoto *et al*. (2018) demonstrated that CTZ 50 mg/kg for seven days reduced interleukin-6 and TNFα [43]. CTZ prevents nitric oxide (NO), prostaglandin E2 (PGE2), cytokines, such as IL1, TNF α, and monocyte chemoattractant protein-1 (MCP-1) production by inhibiting extracellular signal-regulated kinases 1 and 2 (ERK1/2) and c-Jun N-terminal kinase (JNK) [44].

In this study, CTZ 10 mg/kg orally as a prophylactic dose, significantly reduced expression of BAX in relation to AK group. These results agree with a previous study [15] which reported that CTZ 10 mg/kg for eight days produced a significant reduction in BAX expression in gentamycin-induced nephrotoxicity model. CTZ suppresses signals of mitochondria-dependent apoptosis. Additionally, it reduces cytochrome c release from mitochondria and down-regulates BAX expression [45].

This histopathology findings revealed that AK 400 mg/kg was associated with disturbances in the kidney histopathological picture, including inflammatory cell infiltration, tubular epithelial lining degeneration, and tubular necrosis. These results agree with Abdel Fattah and Gaballah, (2020), who demonstrated that marked degenerative changes in the kidney and marked tubular necrosis occurred with AK [46].

EA administration before AK exhibited an improvement in the histopathological changes as it decreased inflammation and necrosis. These results are consistent with Bhattacharjee *et al*., 2021, who stated that EA 25, 50 mg/kg reduced histopathological changes and renal tubular necrosis in lead-induced nephrotoxicity [32]. Also, CTZ administration one hour before AK shows a reduction in inflammation and tubular necrosis. These results agree with Abdelsameea and colleagues [15] who reported that administration of CTZ 10 mg/kg rat eases degenerative changes in the renal cortex in gentamycin-induced nephrotoxicity model.

In this study, oral administration of EA 10 mg/kg plus CTZ 10 mg/kg before AK 400 mg/kg produced a more significant reduction of BUN and creatinine; more significant reduction of oxidation parameter MDA; more significant reduction of antioxidant parameters: GSH, SOD, and CAT; more significant reduction of inflammatory mediators: TNFα, IL6; more significant reduction of NFκB and BAX expression; more improvement in the histopathological changes developed in kidney tissue by AK than each drug alone due to the synergistic effect of both drugs.

## Conclusion

EA and CTZ have a renoprotective effect partially due to their antioxidant, anti-inflammatory, and anti-apoptotic effects.

## Supporting information

S1 Table Effect of ellagic acid, cilostazol and their combination on amikacin induced nephrotoxicity. Data are represented as mean ± SE. AK, amikacin; EA, ellagic acid; CTZ: cilostazol, BUN, blood urea nitrogen; MDA, malondialdehyde; GSH, reduced glutathione; SOD, superoxide dismutase; CAT, catalase; TNFα, tumor necrosis factor-alpha; IL6, interleukin 6, NFκB, nuclear factor kappa B; BAX, bcl-2 associated x protein. Values without common small letters are significantly different.
(DOCX)

## References

[1] Hlail AT, Faraj HR, Abdulredha WS. The protective effect of Omega3 against amikacin-induced nephrotoxicity in rats. Systematic reviews in pharmacy. 2020;11(9):110–7.

[2] Devarajan P. Update on mechanisms of ischemic acute kidney injury. Journal of the American Society of Nephrology. 2006 Jun 1;17(6):1503–20. doi: 10.1681/ASN.2006010017 16707563

[3] Miller RP, Tadagavadi RK, Ramesh G, Reeves WB. Mechanisms of cisplatin nephrotoxicity. Toxins. 2010 Nov;2(11):2490–518. doi: 10.3390/toxins2112490 22069563 PMC3153174

[4] Calvin AD, Misra S, Pflueger A. Contrast-induced acute kidney injury and diabetic nephropathy. Nature reviews. Nephrology. 2010 Nov;6(11):679–88. doi: 10.1038/nrneph.2010.116 20877303 PMC4476402

[5] Abdel-Daim MM, Ahmed A, Ijaz H, Abushouk AI, Ahmed H, Negida A, et al. Influence of Spirulina platensis and ascorbic acid on amikacin-induced nephrotoxicity in rabbits. Environmental science and pollution research international. 2019 Mar;26(8):8080–6. doi: 10.1007/s11356-019-04249-4 30685861

[6] Ozer MK, Bilgic S, Armagan I, Savran M. Thymoquinone protection from amikacin induced renal injury in rats. Biotechnic and histochemistry. 2020 Feb 17;95(2):129–36. doi: 10.1080/10520295.2019.1650957 31502890

[7] Prajapati B, Singha M. Comparative evaluation of the toxicity of amikacin and cefepime on rat’s kidney and liver. International journal of PharmTech research. 2010;3(4):2149–54.

[8] Firdaus F, Zafeer MF, Anis E, Ahmad M, Afzal M. Ellagic acid attenuates arsenic induced neuro-inflammation and mitochondrial dysfunction associated apoptosis. Toxicology reports. 2018 Jan 1;5:411–7. doi: 10.1016/j.toxrep.2018.02.017 29854611 PMC5978009

[9] Ciuclan L, Ehnert S, Ilkavets I, Weng HL, Gaitantzi H, Tsukamoto H, et al. TGF-β enhances alcohol dependent hepatocyte damage via down-regulation of alcohol dehydrogenase I. Journal of hepatology. 2010 Mar 1;52(3):407–16. doi: 10.1016/j.jhep.2009.12.003 20129692

[10] Ragab D, Abdallah DM, El-Abhar HS. Cilostazol renoprotective effect: Modulation of PPAR-γ, NGAL, KIM-1 and IL-18 underlies its novel effect in a model of ischemia-reperfusion. PLOS ONE. 2014 May 9;9(5):e95313. doi: 10.1371/journal.pone.0095313 24816434 PMC4015937

[11] Rondina MT, Weyrich AS. Targeting phosphodiesterases in anti-platelet therapy. Handbook of experimental pharmacology. 2012;(210):225–38. doi: 10.1007/978-3-642-29423-5_9 22918733 PMC3682780

[12] Hafez HM, Ibrahim MA, Zedan MZ, Hassan M, Hassanein H. Nephroprotective effect of cilostazol and verapamil against thioacetamide-induced toxicity in rats may involve Nrf2/HO-1/NQO-1 signaling pathway. Toxicology mechanisms and methods. 2019 Feb 12;29(2):146–52. doi: 10.1080/15376516.2018.1528648 30295103

[13] Parlakpinar H, Ozer MK, Sahna E, Vardi N, Cigremis Y, Acet A. Amikacin-induced acute renal injury in rats: Protective role of melatonin. Journal of pineal research. 2003 Sep;35(2):85–90. doi: 10.1034/j.1600-079x.2003.00059.x 12887650

[14] Sepand MR, Ghahremani MH, Razavi-Azarkhiavi K, Aghsami M, Rajabi J, Keshavarz-Bahaghighat H, et al. Ellagic acid confers protection against gentamicin-induced oxidative damage, mitochondrial dysfunction and apoptosis-related nephrotoxicity. Journal of pharmacy and pharmacology. 2016 Sep;68(9):1222–32. doi: 10.1111/jphp.12589 27364420

[15] Abdelsameea AA, Mohamed AM, Amer MG, Attia SM. Cilostazol attenuates gentamicin-induced nephrotoxicity in rats. Experimental and toxicologic pathology. 2016 Apr 1;68(4):247–53. doi: 10.1016/j.etp.2016.01.002 26809659

[16] Ohkawa H, Ohishi N, Yagi K. Assay for lipid peroxides in animal tissues by thiobarbituric acid reaction. Analytical biochemistry. 1979 Jun 1;95(2):351–8. doi: 10.1016/0003-2697(79)90738-3 36810

[17] Ellman GL. Tissue sulfhydryl groups. Archives of biochemistry and biophysics. 1959 May 1;82(1):70–7. doi: 10.1016/0003-9861(59)90090-6 13650640

[18] Aebi H. Catalase in vitro. Methods in enzymology. 1984 Jan 1;105:121–6. doi: 10.1016/s0076-6879(84)05016-3 6727660

[19] Sun YI, Oberley LW, Li Y. A simple method for clinical assay of superoxide dismutase. Clinical chemistry. 1988 Mar 1;34(3):497–500. 3349599

[20] Bancroft JD, Gamble M Theory and practice of histological techniques 5th edition, eds. Bancroft JD and Gamble M. Churchill Livingstone. 2002.

[21] McWilliam SJ, Antoine DJ, Smyth RL, Pirmohamed M. Aminoglycoside-induced nephrotoxicity in children. Pediatric nephrology. 2017 Nov;32(11):2015–25. doi: 10.1007/s00467-016-3533-z 27848094 PMC5624973

[22] Loschen G, Azzi A, Richter C, Flohé L. Superoxide radicals as precursors of mitochondrial hydrogen peroxide. FEBS letters. 1974 May 15;42(1):68–72. doi: 10.1016/0014-5793(74)80281-4 4859511

[23] Tsikas D. Assessment of lipid peroxidation by measuring malondialdehyde (MDA) and relatives in biological samples: Analytical and biological challenges. Analytical biochemistry. 2017 May 1;524:13–30. doi: 10.1016/j.ab.2016.10.021 27789233

[24] Lushchak VI. Glutathione homeostasis and functions: Potential targets for medical interventions. Journal of amino acids. 2012;2012:736837. doi: 10.1155/2012/736837 22500213 PMC3303626

[25] Wargo KA, Edwards JD. Aminoglycoside-induced nephrotoxicity. Journal of pharmacy practice. 2014 Dec;27(6):573–7. doi: 10.1177/0897190014546836 25199523

[26] Zhou B, Li Q, Wang J, Chen P, Jiang S. Ellagic acid attenuates streptozocin induced diabetic nephropathy via the regulation of oxidative stress and inflammatory signaling. Food and chemical toxicology. 2019 Jan 1;123:16–27. doi: 10.1016/j.fct.2018.10.036 30342113

[27] Ozbek E, Cekmen M, Ilbey YO, Simsek A, Polat EC, Somay A. Atorvastatin prevents gentamicin-induced renal damage in rats through the inhibition of p38-MAPK and NF-kB pathways. Renal failure. 2009 Jan 1;31(5):382–92. doi: 10.1080/08860220902835863 19839839

[28] Helmy A, El-Shazly M, Omar N, Rabeh M, Abdelmohsen UR, Tash R, et al. Increment of lysosomal biogenesis by combined extracts of gum arabic, parsley, and corn silk: A reparative mechanism in mice renal cells. Evidence-based complementary and alternative medicine: eCAM. 2020 Jul 11;2020:8631258. 32733590 10.1155/2020/8631258 PMC7369655

[29] Servais H, Jossin Y, Van Bambeke F, Tulkens PM, Mingeot-Leclercq MP. Gentamicin causes apoptosis at low concentrations in renal LLC-PK1 cells subjected to electroporation. Antimicrobial agents and chemotherapy. 2006 Apr;50(4):1213–21. doi: 10.1128/AAC.50.4.1213-1221.2006 16569831 PMC1426926

[30] Ateşşahín A, Çeríbaşi AO, Yuce A, Bulmus O, Çikim G. Role of ellagic acid against cisplatin‐induced nephrotoxicity and oxidative stress in rats. Basic and clinical pharmacology and toxicology. 2007 Feb;100(2):121–6. doi: 10.1111/j.1742-7843.2006.00015.x 17244261

[31] Nejad KH, Gharib-Naseri MK, Sarkaki A, Dianat M, Badavi M, Farbood Y. Effects of ellagic acid pretreatment on renal functions disturbances induced by global cerebral ischemic-reperfusion in rat. Iranian journal of basic medical sciences. 2017 Jan;20(1):75–82. doi: 10.22038/ijbms.2017.8098 28133528 PMC5243978

[32] Bhattacharjee A, Kulkarni VH, Chakraborty M, Habbu PV, Ray A. Ellagic acid restored lead-induced nephrotoxicity by anti-inflammatory, anti-apoptotic and free radical scavenging activities. Heliyon. 2021 Jan 1;7(1):e05921. doi: 10.1016/j.heliyon.2021.e05921 33490681 PMC7809373

[33] Ríos JL, Giner RM, Marín M, Recio MC. A pharmacological update of ellagic acid. Planta medica. 2018 Oct;84(15):1068–93. doi: 10.1055/a-0633-9492 29847844

[34] ALTamimi JZ, Alshammari GM, AlFaris NA, Alagal RI, Aljabryn DH, Albekairi NA, et al. Ellagic acid protects against non-alcoholic fatty liver disease in streptozotocin-diabetic rats by activating AMPK. Pharmaceutical biology. 2022;60(1):25–37. doi: 10.1080/13880209.2021.1990969 34870551 PMC8654409

[35] Alfei S, Marengo B, Zuccari G. Oxidative stress, antioxidant capabilities, and bioavailability: Ellagic acid or urolithins? Antioxidants. 2020 Aug;9(8):707. doi: 10.3390/antiox9080707 32759749 PMC7465258

[36] Marín M, María Giner RM, Ríos JL, Recio MC. Intestinal anti-inflammatory activity of ellagic acid in the acute and chronic dextrane sulfate sodium models of mice colitis. Journal of ethnopharmacology. 2013 Dec 12;150(3):925–34. doi: 10.1016/j.jep.2013.09.030 24140585

[37] Ghasemi-Niri SF, Maqbool F, Baeeri M, Gholami M, Abdollahi M. Phosalone-induced inflammation and oxidative stress in the colon: Evaluation and treatment. World journal of gastroenterology. 2016 Jun 7;22(21):4999–5011. doi: 10.3748/wjg.v22.i21.4999 27275092 PMC4886375

[38] Cornélio Favarin D, Martins Teixeira M, Lemos de Andrade E, de Freitas Alves C, Lazo Chica JE, Artério Sorgi C, et al. Anti-inflammatory effects of ellagic acid on acute lung injury induced by acid in mice. Mediators of inflammation. 2013;2013:164202. doi: 10.1155/2013/164202 23533300 PMC3600201

[39] Mohammed ET, Hashem KS, Abdelazem AZ, Foda FAMA. Prospective protective effect of ellagic acid as a SIRT1 activator in iron oxide nanoparticle-induced renal damage in rats. Biological trace element research. 2020 Jan 13;198(1):177–88. doi: 10.1007/s12011-020-02034-w 31933277

[40] Yun JM, Chien A, Jialal I, Devaraj S. Resveratrol up-regulates SIRT1 and inhibits cellular oxidative stress in the diabetic milieu: Mechanistic insights. The journal of nutritional biochemistry. 2012 Jul 1;23(7):699–705. doi: 10.1016/j.jnutbio.2011.03.012 21813271 PMC3209497

[41] Gokce M, Yuzbasioglu MF, Bulbuloglu E, Oksuz H, Yormaz S, Altınoren O, et al. Cilostazol and diltiazem attenuate cyclosporine-induced nephrotoxicity in rats. InTransplantation proceedings. 2012 Jul 1 (Vol. 44, No. 6, pp. 1738–1742). Elsevier. doi: 10.1016/j.transproceed.2012.04.025 22841259

[42] Hermes Tde A, Macedo AB, Fogaça AR, Moraes LH, de Faria FM, Kido LA, et al. Beneficial cilostazol therapeutic effects in mdx dystrophic skeletal muscle. Clinical and experimental pharmacology and physiology. 2016 Feb;43(2):259–67. doi: 10.1111/1440-1681.12521 26639107

[43] Sakamoto T, Ohashi W, Tomita K, Hattori K, Matsuda N, Hattori Y. Anti-inflammatory properties of cilostazol: Its interruption of DNA binding activity of NF-κB from the toll-like receptor signaling pathways. International immunopharmacology. 2018 Sep 1;62:120–31. doi: 10.1016/j.intimp.2018.06.021 30005227

[44] Jung WK, Lee DY, Park C, Choi YH, Choi I, Park SG, et al. Cilostazol is anti‐inflammatory in BV2 microglial cells by inactivating nuclear factor‐kappaB and inhibiting mitogen-activated protein kinases. British journal of pharmacology. 2010 Mar;159(6):1274–85. doi: 10.1111/j.1476-5381.2009.00615.x 20128801 PMC2848931

[45] Park SY, Bae JU, Hong KW, Kim CD. HO-1 induced by cilostazol protects against TNF-α-associated cytotoxicity via a PPAR-γ-dependent pathway in human endothelial cells. The Korean journal of physiology and pharmacology. 2011 Apr 1;15(2):83–8. doi: 10.4196/kjpp.2011.15.2.83 21660147 PMC3104202

[46] Badr K, Abdel Fattah A, Gaballah I. Possible Protective Potential of atorvastatin and Black Seed (Nigella sativa) Oil in amikacin induced Nephrotoxicity in Adult Male albino Rats. The Egyptian Journal of Forensic Sciences and Applied Toxicology. 2020 Sep 1;20(3):55–65.

