# Supplementary material for: Ellagic acid and cilostazol ameliorate amikacin-induced nephrotoxicity in rats by downregulating oxidative stress, inflammation, and apoptosis
Source: PLoS One. 2022 Jul 18;17(7):e0271591. doi: 10.1371/journal.pone.0271591 (PMC9292089; doi:10.1371/journal.pone.0271591)
Supplement: S1 Table — Data are represented as mean ± SE. AK, amikacin; EA, ellagic acid; CTZ: cilostazol, BUN, blood urea nitrogen; MDA, malondialdehyde; GSH, reduced glutathione; SOD, superoxide dismutase; CAT, catalase; TNFα, tumor necrosis factor-alpha; IL6, interleukin 6, NFκB, nuclear factor kappa B; BAX, bcl-2 associated x protein. Values without common small letters are significantly different. (DOCX) [file pone.0271591.s001.docx]

| AK+  EA+CTZ | AK+  CTZ | AK+  EA | CTZ  10mg/kg | EA  10mg/kg | | AK  400mg/kg | Control  normal | Groups |
| --- | --- | --- | --- | --- | --- | --- | --- | --- |
| 28.6  ±2.3  a | 40.56  ±1.9  c | 38.54 ± 2.56  c | 29.1  ±1.2  a | 27.5 ± 1.5  a | | 85.8  ±2.8 b | 28.59  ±1.7  a | **BUN**  **(mg/dl)** |
| 0.3  ±0.04  a | 0.61  ±0.02  c | 0.56  ±0.02  c | 0.23  ±0.05  a | 0.21  ±.02  a | | 1.8  ±0.26 b | 0.22  ±0.03  a | **Creatinine**  **(mg/dl)** |
| 40.61  ±2.71  d | 68.61  ±3.32  c | 61.83 ±4.6  c | 25.5  ±3.2  a | | 24.11  ±2.5  a | 107.9  ±4.58  b | 25.13  ±2.74  a | **MDA (nmol/gm)** |
| 50.83  ±3.14  d | 43.04  ±2.1  c | 44.74 ±4.5  c | 63.2  ±2.1  a | | 62.5  ±3.2  a | 24.51 ±2.5  b | 64.54  ±2.18  a | **GSH (nmol/gm)** |
| 8.24  ±0.27  a | 5.91 ±0.16  c | 6.72 ±0.24  c | 10.4  ±1.1  a | | 9.50  ±0.5  a | 2.29 ±0.21  b | 10.04  ±0.54  a | **SOD (u/gm)** |
| 110.34  ±6.6  a | 84.3  ±2.28  c | 90.59  ±2.7  c | 121.3  ±3.4  a | | 118  ±2.5  a | 63.95 ±4.8  b | 120 ±1.72  a | **CAT (u/gm)** |
| 34.56  ±1.77  d | 51.2  ±2.02  c | 49.2  ±1.13  c | 16.2  ±2.1  a | | 15.33  ±1.2  a | 104.4  ±5  b | 15.79  ±1.6  a | **TNFα (Pg/mg,pt)** |
| 36.7  ±3.84  a | 73.44  ±2.17  c | 65.53  ±3.44  c | 33.8  ±1.5  a | | 32  ±1.2  a | 126.2  ±2.5  b | 33.6  ±2.06  a | **IL6 (Pg/mg,pt)** |
| 1.56  ±0.07  a | 2.76  ±0.08  c | 2.66 ±0.13  c | 1.02  ±0.003  a | | 1  ±0.003  a | 6.24 ±0.11  b | 1.02  ±0.005  a | **NFκB** |
| 1.77  ±0.16  d | 4.12  ±0.07  c | 3.69 ±0.17  c | 1.02  ±0.002  a | | 1.01  ±002  a | 7.7  ±0.37  b | 1.02 ±0.004  a | **BAX** |

Data are represented as mean ± SE

**AK**, amikacin; **EA**, ellagic acid; **CTZ**: cilostazol, **BUN**, blood urea

nitrogen; **MDA**, malondialdehyde; **GSH**, reduced glutathione; **SOD**,

superoxide dismutase; **CAT**, catalase; **TNFα**, tumor necrosis factor-

alpha; **IL6**, interleukin 6, **NFκB**, nuclear factor kappa B; **BAX**, bcl-2

associated x protein.

Values without common small letters are significantly different
